# Spanish Version of the Teachers’ Sense of Efficacy Scale: An Adaptation and Validation Study

**DOI:** 10.3389/fpsyg.2021.714145

**Published:** 2021-11-11

**Authors:** Fátima Salas-Rodríguez, Sonia Lara, Martín Martínez

**Affiliations:** ^1^Department of Learning and Curriculum, University of Navarra, Pamplona, Spain; ^2^Cognitive and Affective Methods in Psychology, Department of Research Theory and Methods in Education and Psychology, University of Navarra, Pamplona, Spain

**Keywords:** Teachers’ Sense of Efficacy Scale, construct validity, criterion validity, self-efficacy, job satisfaction, Mexico

## Abstract

The Teachers’ Sense of Efficacy Scale (TSES) has been the most widely used instrument to assess teacher efficacy beliefs. However, no study has been carried out concerning the TSES psychometric properties with teachers in Mexico, the country with the highest number of Spanish-speakers worldwide. The purpose of the present study is to examine the reliability, internal and external validity evidence of the TSES (short form) adapted into Spanish with a sample of 190 primary and secondary Mexican teachers from 25 private schools. Results of construct analysis confirm the three-factor-correlated structure of the original scale. Criterion validity evidence was established between self-efficacy and job satisfaction. Differences in self-efficacy were related to teachers’ gender, years of experience and grade level taught. Some limitations are discussed, and future research directions are recommended.

## Introduction

Teacher self-efficacy is critical to the creation of effective teaching and learning environments. At first, the interest in this variable was due to the relationship found between teacher efficacy beliefs, motivation, and academic improvement of their students (Armor et al., 1976; Berman et al., 1977). However, in recent years, teacher self-efficacy has taken on special importance due to its relationship with teachers’ well-being. Previous studies have found that teachers with high levels of self-efficacy tend to have higher levels of job satisfaction (Caprara et al., 2006; Skaalvik and Skaalvik, 2010; Vieluf et al., 2013; Malinen and Savolainen, 2016; Burić and Kim, 2021), lower levels of stress (Klassen and Chiu, 2010; Zee and Koomen, 2016; Skaalvik and Skaalvik, 2017; Fathi et al., 2021), and less desire to leave the profession (Chesnut and Burley, 2015; Wang et al., 2015; Skaalvik and Skaalvik, 2016).

Studies such as those of Klassen et al. (2009), Ruan et al. (2015), and Fackler (2018) have demonstrated that teacher self-efficacy is a valid construct in different cultural contexts. Nonetheless, there is a lack of research on this subject in Spanish-speaking countries. Casas Moreno and Blanco-Blanco (2016) asserts that the scarcity of studies in these countries may be due to a lack of psychometric tools that allow a valid and reliable assessment of teachers’ efficacy beliefs.

This research seeks to promote the study of self-efficacy in Spanish-speaking contexts by adapting the Teachers’ Sense of Efficacy Scale (TSES; Tschannen-Moran and Woolfolk Hoy, 2001) into Spanish and studying its psychometric properties with a sample of Mexican teachers.

### Teachers’ Self-Efficacy: Its Meaning and Measurement

Framed in Bandura’s social cognitive theory, teacher self-efficacy is defined as “the teacher’s belief in his or her capability to organize and execute courses of action required to successfully accomplish a specific teaching task in a particular context” (Tschannen-Moran et al., 1998, p. 233). Teachers with high levels of self-efficacy beliefs are distinguished by having a greater commitment to the teaching profession and its students (Chesnut and Burley, 2015); showing greater openness to change and innovation (Bandura, 1997; Tsigilis et al., 2007); spending more time teaching in class (Organization for Economic Co-operation and Development [OECD], 2019a); having a more positive affect (Burić and Moè, 2020); presenting greater levels of instructional quality (Künsting et al., 2016; Burić and Kim, 2020); tending to collaborate to a greater extent with other teachers (Skaalvik and Skaalvik, 2010); and establishing closer relationships with their students (Zee and Koomen, 2017; Hajovsky et al., 2020; Wettstein et al., 2021).

Although teacher self-efficacy has always been related to educational improvement, one of the problems in the study of these beliefs has been reaching a consensus regarding the conceptualization and evaluation of this construct (Skaalvik and Skaalvik, 2007). To address this, Tschannen-Moran et al. (1998) conducted a review of the theoretical and empirical foundations of teacher self-efficacy and proposed an integrated model based on the idea that in order to properly asses teachers’ self-efficacy beliefs, two factors need to be known: the assessment of one’s own competence and the analysis of the teaching task. Essentially, teachers develop their self-efficacy beliefs based on the assessment of their own competence with respect to the perceived demands of the teaching task at hand (Tschannen-Moran et al., 1998). In this way, teachers anticipate the difficulty of the task and assess the resources at their disposal by analyzing the contextual factors that favor or hinder teaching performance. Perception of one’s own teaching competence is related to the tasks to be fulfilled, i.e., how capable I am to achieve a certain performance with respect to a particular task.

This integrated model became the basis for the TSES (Tschannen-Moran and Woolfolk Hoy, 2001). The TSES measures teacher self-efficacy using 24 items in its long form or 12 items in its short form. Items are grouped into three different but inter-correlated factors: efficacy for instructional strategies, efficacy for classroom management, and efficacy for student engagement. These three factors reflect the multi-faceted nature of self-efficacy both by presenting a wide variety of teaching tasks and maintaining a balance between the general and the specific, which allows the scale to be used in different contexts and at different educational levels. Items focus on assessing teachers’ judgment of their own capability by including the stem, “How much can you do to…” or “To what extent can you…” and are measured following a nine-point Likert scale.

Evidence on the TSES was first validated with a convenience sample of 255 in-service and 103 pre-service teachers from the United States. After using principal-axis factoring with Varimax rotation, a three-factor structure was found for the in-service teachers, and a single factor was recommended for the pre-service teachers. Thanks to its solid theoretical foundation and its stable factor structure, the TSES has been by far the most widely used scale for assessing teachers’ self-efficacy beliefs (Woolfolk Hoy and Spero, 2005). Multiple studies have adapted and evaluated validity evidence of the scale with in-service and pre-service teachers of different countries and languages; Table 1 presents some of these studies. As a result, a more comprehensive understanding of the construct and the TSES has been obtained.

**TABLE 1:** Psychometric studies of the TSES around the world.

**Article**	**Sampling**	**Participants**	**Country**	**Scale form**	**Analyses**	**Proposed model**	**Fit statistics**
Tschannen-Moran and Woolfolk Hoy, 2001	Convenience	103 pre-service teachers and 255 teachers: preschool (5%), elementary (37%), middle school (29%), high school (29%)	United States	24 and 12 items; 1–9 Likert scale	EFA	Three-factor-correlated model for in-service teachers; one-factor model for pre-service teachers	–
Burgueño et al., 2019	Convenience	358 pre-service teachers	Spain	24, 12, and 11 items; 1–9 Likert scale	CFA	Modified three-factor-correlated model for the 11 items scale form	χ^2^/*df* = 1.7; CFI = 0.979; TLI = 0.972; SRMR = 0.031; RMSEA = 0.052
Çapa et al., 2005	Convenience	628 pre-service teachers	Turkey	24 items; 1–9 Likert scale	CFA	Three-factor-correlated model for the 24 items scale form	CFI = 0.99; TLI = 0.99; RMSEA = 0.065
Covarrubias Apablaza and Mendoza Lira, 2016	Convenience	544 teachers: kindergarten (9.2%), primary (52.2%), secondary (38.6%)	Chile	24 items; 1–5 Likert scale	EFA/CFA	Four-factor model for the 17 items scale form	χ^2^/*df* = 1.36; CFI = 0.959; IFI = 0.96; SRMR = 0.05; RMSEA = 0.054
Dominguez-Lara et al., 2019	NS	347 in-service teachers	Peru	24 items; 1–5 Likert scale	CFA/ESEM	One-factor model for the 24 items scale form	CFI = 0.949; RMSEA = 0.065
Htang, 2018	Convenience	101 pre-service teachers	Myanmar	12 items; 1–5 Likert scale	EFA/CFA	Three-factor-correlated model for the 12 items scale form	χ^2^/*df* = 1.36; CFI = 0.966; IFI = 0.967; SRMR = 0.054; RMSEA = 0.06
Khairani and Makara, 2020	Purposive	122 pre-service teachers and 191 secondary teachers	Malaysia	24 items; 1–5 Likert scale	CFA	Modified three-factor-correlated model for the 21 items scale form	χ^2^/*df* = 2.35; CFI = 0.883; TLI = 0.87; SRMR = 0.054; RMSEA = 0.066
Koniewski, 2019	Two-stage cluster	4465 teachers: primary (46.6%), secondary (53.4%)	Poland	24 items; 1–9 Likert scale	CFA	Modified three-factor-correlated model for the 24 items scale form	CFI = 0.908–0.924; SRMR = 0.043–0.053; RMSEA = 0.044–0.053
Ninković and Knežević-Florić, 2018	Convenience	452 teachers: primary (20.8%), secondary (33.8%), high school (45.4%)	Serbia	12 items; 1–9 Likert scale	CFA	Modified three-factor-correlated model for the 12 items scale form	χ^2^/*df* = 2.84; CFI = 0.953; SRMR = 0.04; RMSEA = 0.074
Tsigilis et al., 2010	Convenience	405 primary and secondary teachers	Greece	24 items; 1–9 Likert scale	CFA	Three-factor-correlated model for the 24 items scale form	χ^2^/*df* = 4.06; CFI = 0.893; SRMR = 0.058; RMSEA = 0.088
Valls et al., 2020	NS	283 teachers: primary (59.7%), secondary (35%), combined (5.3%)	Switzerland	12 items; 1–9 Likert scale	CFA	Three-factor-correlated model for the 12 items scale form	χ^2^/*df* = 3.63; CFI = 0.90; TLI = 0.87; SRMR = 0.061; RMSEA = 0.097

*CFA, confirmatory factor analysis; EFA, exploratory factor analysis; ESEM, exploratory structural equation modeling; NS, not specified; RMSEA, root mean square error of approximation; SRMR, standardized root mean square residual; CFI, comparative fit index; TLI, Tucker-Lewis index.*

### Teachers’ Sense of Efficacy Scale From an International Perspective

Regarding the psychometric properties of the TSES, prior investigations suggest the scale has been characterized by strong levels of reliability across diverse cultural contexts (Klassen et al., 2009; Ruan et al., 2015). Construct validity evidence has been mainly studied through confirmatory factor analysis (CFA), and the three correlated factor structure proposed by Tschannen-Moran and Woolfolk Hoy (2001) has been supported by several studies (see Table 1). However, in order to improve the goodness-of-fit of the three-factor model, certain studies have allowed some items’ errors to correlate (Klassen et al., 2009; Ninković and Knežević-Florić, 2018) or have removed items with low loadings or cross-loaded with other factors (Tsigilis et al., 2010; Ruan et al., 2015; Khairani and Makara, 2020).

Regarding criterion validity evidence, teacher self-efficacy, as measured by the TSES, has been previously related to job satisfaction (Klassen et al., 2009; Tsigilis et al., 2010; Ninković and Knežević-Florić, 2018), being a main determinant of this variable and influencing teachers’ attitudes and performance (Caprara et al., 2003). Specifically, teachers with high levels of self-efficacy tend to demonstrate higher levels of job satisfaction (Caprara et al., 2006; Klassen and Chiu, 2010; Vieluf et al., 2013).

In the Latin American context, the psychometric properties of the TSES have been less studied, and the few existing studies present mixed results. For instance, with a sample of in-service Chilean teachers, Covarrubias Apablaza and Mendoza Lira (2016) reduced the scale from 24 to 17 items and grouped them by four different factors: the three original factors and a new one named *efficacy in attending to the uniqueness of students*. On the other hand, Dominguez-Lara et al. (2019) used the TSES (long version) with a sample of in-service Peruvian teachers and found that the one-factor model adjusted better to the collected data. Further studies are needed in order to ascertain how the TSES behaves in the Latin American context.

According to previous cross-cultural studies, teacher self-efficacy has a similar meaning in different countries (Vieluf et al., 2013; Ruan et al., 2015; Fackler and Malmberg, 2016). Nonetheless, self-efficacy beliefs have been known to be context-dependent and may vary according to cultural values and teacher demographic variables such as gender, teaching field, and teaching experience (Dilekli and Tezci, 2020). Therefore, it is necessary to take into account the characteristics of the sample while studying teacher self-efficacy to better understand the results obtained in the Latin American context.

### Present Study

The overall purpose of this study is to obtain internal and external validity evidence supporting the use of the Teacher’s Sense of Self-Efficacy Scale (TSES, Tschannen-Moran and Woolfolk Hoy, 2001) in Spanish speakers while it is tested on a sample of private school teachers in Mexico. To achieve this purpose, we set out to determine if the three-dimensional factor-analytic solution presented by the original short form of the TSES is replicated in a sample of Mexican teachers, and the extent to which the TSES subscales are related in theoretically meaningful ways to subscales of job satisfaction and other demographic variables such as teacher’s gender, subject taught, school level, teaching experience, and school model. Internal validity evidence will support that Mexican teachers develop capabilities on instructional strategies, classroom management, and student engagement; that the content domain of the test is consistent with self-efficacy perception; that test scores can be generalized across sets of items; that the level of self-efficacy in Mexican teachers can be validly assessed; and that teachers with high scores on the test will have a higher perception of self-efficacy than teachers with low scores.

Criterion validity evidence will support that teachers’ self-efficacy beliefs will be significantly and directly associated with job satisfaction in this sample. In this regard, previous studies have found weak correlations between these two variables. For example, Ninković and Knežević-Florić (2018) found that job satisfaction correlated with each subscale of the TSES r_*IS*_ = 0.40, r_*SE*_ = 0.46, r_*CM*_ = 0.38. Similarly, Klassen et al. (2009) found these significant correlations in teachers from Canada, Cyprus, South Korea, and the United States, ranging from 0.17 to 0.48.

Regarding teachers’ demographic variables, previous studies have shown that variables such as gender, school grade, and teaching experience predict teachers’ self-efficacy (Klassen and Chiu, 2010; Perera et al., 2019). While studies on the differences in teacher self-efficacy according to teacher gender have yielded inconsistent results, it is expected that female and male teachers present different levels of self-efficacy (Lumpe et al., 2012; Perera et al., 2019). In accordance with school grade, the literature has shown that primary school teachers tend to have higher levels of self-efficacy than secondary teachers (Fives and Buehl, 2009). Finally, teacher’s self-efficacy has shown a non-linear relationship with teaching experience (Klassen and Chiu, 2010); in which self-efficacy increases from 0 to about 20 years of experience and then declines as years of experience increase.

Our hypotheses are as follows:

H1. The TSES Spanish version will have the same factor structure as the original scale for in-service teachers (Tschannen-Moran and Woolfolk Hoy, 2001): three correlated factors—Instructional strategies, Classroom management, Student engagement—with four items each. Internal validity evidence will be examined by conducting a CFA to analyze whether the items load on their original factor.

H2. Teacher self-efficacy will present a positive and significant weak correlation with teachers’ job satisfaction, as observed in multiple studies (Caprara et al., 2003; Klassen et al., 2009; Skaalvik and Skaalvik, 2010; Tsigilis et al., 2010; Vieluf et al., 2013; Ninković and Knežević-Florić, 2018). Therefore, we expect to find significant correlation between both scales -TSES and JSC- and their factors. This hypothesized correlation will provide external validity evidence to the TSES scale in the Mexican sample.

H3. Considering previous studies, teacher self-efficacy and its factors will present significant differences regarding demographic variables.
H3.1 Teachers’ levels of self-efficacy will differ significantly according to gender.
H3.2 Primary school teachers will have higher levels of self-efficacy than secondary school teachers.
H3.3 Years of experience will affect self-efficacy of teachers; self-efficacy will increase during the first years of the career but will decrease during later stages.

Testing these hypotheses will help us understand how teacher self-efficacy behaves within the Mexican sample, shedding light on possible avenues of action.

## Materials and Methods

### Participants and Procedure

The convenience sample for this study consisted of 190 in-service teachers (120 females, 70 males; *M*_*age*_ = 40.89, *SD* = 10.05) from 25 private schools in Mexico. Among the teachers from whom the data were collected, 47.9% taught Spanish and 52.1% taught mathematics, to students in 4th grade (*n* = 45), 5th grade (*n* = 39), 6th grade (*n* = 34), 7th grade (*n* = 34), and 8th grade (*n* = 38). Years of teaching experience ranged from 1 to 41, with a mean of 16 years (*SD* = 9.98).

It is important to underline why we selected in-service teachers from private schools in Mexico. As shown in Table 1, the TSES has been mostly adapted and validated with primary and secondary teachers. However, in the Mexican context, public schools are not K-12, being private schools the only way to compare self-efficacy beliefs within teachers from the same school but different educational levels.

Data were collected at the end of the 2019–2020 academic year using a Google Forms questionnaire. Participants were aware of the purpose of the study and completed the questionnaire anonymously. Ethical approval was obtained by the Research Ethics Committee of the authors’ affiliated university (Project ID: 2020.042).

### Instrumentation

*Teacher self-efficacy* was measured using the TSES short form (Tschannen-Moran and Woolfolk Hoy, 2001) with the permission of one of the authors of the scale (MTM). This instrument comprises 12 items grouped into three subscales: Efficacy for instructional strategies (IS; four items), Efficacy for classroom management (CM; four items), and Efficacy for student engagement (SE; four items). Items were measured following a nine-point Likert scale, ranging from 1 (*nothing*) to 9 (*a great deal*), and the global score of the TSES was obtained by averaging the mean score of the three factors. The overall reliability of the original scale was good (α = 0.90), as was the consistency of its subscales, with values ranging from 0.81 to 0.86.

The Spanish version of the TSES was established using the translation and back-translation procedure. First, a native Spanish-speaking scholar translated the TSES into Spanish. Second, a native English professional re-translated the scale from Spanish to English. Third, the authors and the professionals reviewed both versions item by item in order to detect semantic and/or conceptual differences between the original and translated versions. Any differences were discussed, and a consensus was reached for each item. Finally, a Mexican scholar and two Mexican education professionals revised the TSES Spanish version to ensure the neutrality of the vocabulary used in the adaptation. It was concluded that no further changes were necessary due to the standard register of the language used in the scale. Table 2 shows the TSES Spanish version.

**TABLE 2:** Spanish and original versions of the Teachers’ Sense of Efficacy Scale (TSES).

**TSES Spanish version**									**Original TSES (Tschannen-Moran and Woolfolk Hoy, 2001)**								
**1**	**2**	**3**	**4**	**5**	**6**	**7**	**8**	**9**	**1**	**2**	**3**	**4**	**5**	**6**	**7**	**8**	**9**
**Nada/En absoluto**	**Muy poco**			**Algo/En alguna medida**	**Bastante**			**Mucho/Muy bien**	**None at all**	**Very little**			**Some degree**	**Quite a bit**			**A great deal**

*Eficacia para las estrategias instruccionales*									*Efficacy for instructional strategies*								

5. ¿En qué medida puedes formular buenas preguntas a tus alumnos?									To what extent can you craft good questions for your students?								
9. ¿En qué medida puedes emplear estrategias de evaluación variadas?									To what extent can you use a variety of assessment strategies?								
10. ¿En qué medida puedes proporcionar explicaciones o ejemplos alternativos cuando tus alumnos tienen dudas?									To what extent can you provide an alternative explanation or example when students are confused?								
12. ¿Hasta qué punto puedes llevar a la práctica estrategias docentes alternativas en el aula?									How well can you implement alternative strategies in your classroom?								

*Eficacia para el manejo de la clase*									*Efficacy for classroom management*								

1. ¿Cuánto puedes hacer para controlar el comportamiento disruptivo en el aula?									How much can you do to control disruptive behavior in classroom?								
3. ¿Cuánto puedes hacer para calmar a un alumno que se comporta de manera disruptiva o ruidosa?									How much can you do to calm a student who is disruptive or noisy?								
6. ¿Cuánto puedes hacer para que tus alumnos cumplan las normas en el aula?									How much can you do to get children to follow classroom rules?								
8. ¿Hasta qué punto puedes establecer un sistema de gestión del aula con cada grupo de alumnos?									How well can you establish a classroom management system with each group of students?								

*Eficacia para la participación de los alumnos*									*Efficacy for student engagement*								

2. ¿Cuánto puedes hacer para motivar a los alumnos que muestran un bajo interés en sus tareas escolares?									How much can you do to motivate students who show low interest in schoolwork?								
4. ¿Cuánto puedes hacer para ayudar a tus alumnos a valorar el aprendizaje?									How much can you do to help your students value learning?								
7. ¿Cuánto puedes hacer para que tus alumnos se crean capaces de realizar con éxito sus tareas escolares?									How much can you do to get students to believe they can do well in schoolwork?								
11. ¿Cuánto puedes apoyar a las familias para que ayuden a sus hijos a ir bien en el colegio?									How much can you assist families in helping their children do well in school?								

*Job satisfaction* was evaluated with the Mexican version of the Job Satisfaction composite scale (JSC) from TALIS (Organization for Economic Co-operation and Development [OECD], 2019b). Two subscales form this scale: Job satisfaction with work environment (JSWE; four items) and Job satisfaction with profession (JSP; four items). Items were coded using a four-point Likert scale ranging from 1 (*strongly disagree*) to 4 (*strongly agree*). The overall reliability of this TALIS scale used in Mexico was good (α = 0.79); however, the internal consistency for its subscales displayed lower, though still acceptable values (ω_*JSWE*_ = 0.75; ω_*JSP*_ = 0.64; Organization for Economic Co-operation and Development [OECD], 2019b).

### Data Analysis

The collected data were analyzed using STATA 13. Prior to conducting the CFAs, the suitability of the sample data was tested. The Kaiser-Meyer-Olkin (KMO) test and Bartlett’s test of sphericity were conducted to verify the adequacy of the data. Secondly, CFAs within the structural equation modeling framework (Brown and Moore, 2012) were applied to evaluate the structural validity evidence of the TSES. Two different models were generated so as to choose the one that better fit the data. First, a one-factor model was tested with all items loading on the same latent factor: teachers’ self-efficacy. Then, a three-factor-correlated model, in which the three latent variables were those proposed by Tschannen-Moran and Woolfolk Hoy (2001): instructional strategies, classroom management, and student engagement.

To compute the quality of the CFAs, the goodness-of-fit of the examined models was tested through different fit indices: χ^2^/*df* ratio, where a ratio ≤3 indicates a good fit (Byrne, 2004); root mean square error of approximation (RMSEA), where a value ≤0.08 suggests a well-fitting model; standardized root mean square residual (SRMR), with acceptable values considered as ≤0.08 (Hu and Bentler, 1999); and comparative fit index (CFI) and Tucker-Lewis index (TLI), where values ≥0.9 demonstrate adequate fit (Bentler, 1990). The internal consistency of the obtained factors and the scale was verified by means of the Cronbach alpha’s coefficient.

Criterion validity evidence of the TSES was assessed using Spearman’s correlation analysis between self-efficacy and job satisfaction. This non-parametric correlation test was used since the data were previously checked for normality with the Shapiro-Wilk test and a non-normal distribution was found. Lastly, the Mann-Whitney *U* test and the Kruskal-Wallis *H* test were used to compare levels of self-efficacy regarding teachers’ demographic variables: teachers’ gender, subject taught, school level, years of teaching experience, and school model. If any test showed significant group differences (*p* < 0.05), a Mann-Whitney *U post hoc* test was performed to compare two groups at a time (corrected for multiple comparisons by Dunn’s Test). The estimated sizes of statistically significant effects are reported through Cohen’s *r*.

## Results

### Construct Validity

The Kaiser-Meyer-Olkin measure and Bartlett’s test of sphericity demonstrated high strength in the relationships among items (KMO = 0.915; χ^2^ = 1172.32, *p* < 0.001), indicating appropriateness to perform a CFA. While the one-factor model showed a poor goodness-of-fit (χ^2^/*df* = 3.53, RMSEA = 0.115, SRMR = 0.059, CFI = 0.881, TLI = 0.854), the three-factor-correlated model showed a significant improvement (χ^2^/*df* = 2.97, RMSEA = 0.102, SRMR = 0.053, CFI = 0.912, TLI = 0.886). However, RMSEA value was higher than recommended thresholds and TLI just below the cutoff score of 0.90.

**FIGURE 1:**
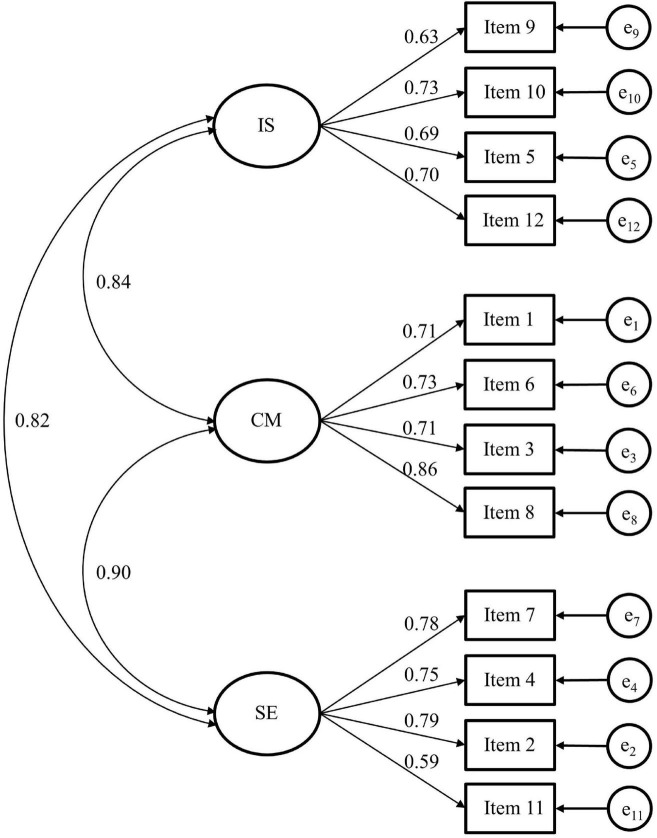
Factor structure of the TSES (Spanish version). The number of the items corresponds to the order followed in the Spanish TSES (see Table 2). IS, efficacy for instructional strategies; CM, efficacy for classroom management; SE, efficacy for student engagement.

The factor structure of the three-factor-correlated model is shown in Figure 1. All items’ factor loadings were higher than 0.30 and showed an excellent loading across their target factor, ranging between 0.59 and 0.86 (*p* < 0.001). Interfactor correlations were strong and positive, particularly the correlation between SE and CM (0.90).

Regarding reliability, the Cronbach’s alpha coefficient of the Spanish TSES was 0.91, while the internal consistencies of its subscales were α_*IS*_ = 0.78, α_*CM*_ = 0.85, α_*SE*_ = 0.82. Thus, internal consistency of the overall TSES was good, while its subscales were acceptable to good. Table 3 presents the descriptive statistics of the Spanish TSES and its subscales, as well as the scores obtained in the original study regarding the short form scale (Tschannen-Moran and Woolfolk Hoy, 2001). The Spanish version of the TSES is presented in Supplementary Annex 1.

**TABLE 3:** Descriptive statistics and reliability for the Spanish and original versions of the TSES.

	**Spanish TSES**					**Original TSES (Tschannen-Moran and Woolfolk Hoy, 2001)**		
	**Mean**	**SD**	**Median**	**IQR**	**α**	**Mean**	**SD**	**α**
TSES	8.1	0.69	8.17	0.83	0.91	7.1	0.98	0.90
IS	8.1	0.72	8.25	1	0.78	7.3	1.2	0.86
CM	8.1	0.78	8.25	1	0.85	6.7	1.2	0.86
SE	8	0.81	8	1.25	0.82	7.2	1.2	0.81

*Since the TSES scores were non-normally distributed in the Spanish TSES, the median has been reported as a central tendency measure and the IQR as a measure of statistical dispersion. TSES, Teachers’ Sense of Efficacy Scale; IS, efficacy for instructional strategies; CM, efficacy for classroom management; SE, efficacy for student engagement; IQR, interquartile range; alpha, Cronbach’ coefficient.*

### Criterion Validity

To verify the evidence of criterion validity for the TSES, correlation analyses were conducted between the variables of teacher self-efficacy and job satisfaction. Prior to these analyses, the Shapiro-Wilk test was used, and a significant deviation from normality was found (*W* = 0.86, *p* < 0.001). Results from the non-parametric Spearman correlation test are shown in Table 4. Not all factors from the TSES were significantly correlated with the JSC scale and subscales. For example, while the JSWE subscale showed a weak correlation with each of the TSES factors, the JSP subscale did not correlate with any of the TSES factors.

**TABLE 4:** Pair-wise correlations between the TSES and JSC.

	**TSES**	**IS**	**CM**	**SE**	**JSC**	**JSWE**	**JSP**
1. TSES	1						
2. IS	0.87***	1					
3. CM	0.91***	0.70***	1				
4. SE	0.89***	0.66***	0.72***	1			
5. JSC	0.19**	0.16*	0.21**	0.14	1		
6. JSWE	0.25***	0.21**	0.26***	0.19**	0.83***	1	
7. JSP	0.09	0.09	0.09	0.06	0.86***	0.49***	1

TSES, Teachers’ Sense of Efficacy Scale; IS, efficacy for instructional strategies; CM, efficacy for classroom management; SE, efficacy for student engagement; JSC, Job satisfaction composite; JSWE, Job satisfaction with work environment; JSP, Job satisfaction with profession; *p < 0.05; ** *p* < 0.01; *** *p* < 0.001.

In this study, the internal consistency for the JSC scale and subscales were good, with acceptable to high alpha and McDonald’s omega coefficients (α_*JSC*_ = 0.79; ω_*JSWE*_ = 0.84; ω_*JSP*_ = 0.63). The Spanish JSC scale is presented in Supplementary Annex 2.

### Group Differences in Teacher Self-Efficacy

Due to the non-normal distribution of the data, the Mann-Whitney *U* test was used to determine if there were significant differences between teachers’ self-efficacy according to their gender and teaching subject (Spanish or mathematics). Results revealed that the male participants had significantly greater levels of self-efficacy than their female colleagues (*z* = 2, *p* = 0.045, *r* = 0.15). However, no significant differences were found between female and male teachers regarding IS, CM, and SE subscales. Regarding differences according to the subject taught, Spanish and math teachers appeared to have similar ranks for teacher self-efficacy, as shown in Table 5.

**TABLE 5:** Medians and IQRs of teacher self-efficacy regarding participants’ demographic variables.

**Variable**		**N**	**TSES**	**IS**	**CM**	**SE**	**TSES comparison *p*-value**
Gender	Female	120	8 (0.92)	8 (1)	8 (1.13)	8 (1.25)	0.046
	Male	70	8.33 (0.92)	8.25 (1)	8.5 (1)	8.25 (1)	

Subject	Spanish	91	8.17 (0.91)	8 (0.75)	8 (1)	8 (1)	0.067
	Mathematics	99	8.17 (0.83)	8.25 (1.25)	8.25 (1)	8.25 (1)	

School grade	4th grade	45	8.17 (0.91)	8 (1.25)	8.25 (1)	8.25 (1)	0.164
	5th grade	39	8.17 (1)	8.25 (1)	8 (1.25)	8.25 (1.25)	
	6th grade	34	8.25 (0.75)	8.13 (1)	8.25 (1)	8.25 (0.75)	
	7th grade	34	7.92 (0.58)	8 (0.75)	7.88 (1)	7.75 (0.5)	
	8th grade	38	8.25 (1.08)	8.38 (1)	8.38 (1)	8.25 (1.25)	

Teaching experience	1–5 years	31	8 (1)	8 (1)	8 (1)	8.25 (1)	0.150
	6–10 years	35	7.83 (0.92)	8 (1)	8 (1.25)	7.75 (1)	
	11–15 years	40	7.88 (1.21)	8.13 (1.13)	7.88 (1.13)	8.13 (1.25)	
	16–20 years	31	8.33 (0.91)	8.25 (1.25)	8.5 (0.75)	8.25 (1.5)	
	21 or more	53	8.25 (0.58)	8.25 (0.75)	8.25 (0.75)	8.25 (0.75)	

School model	Co-education	93	8.17 (0.75)	8.25 (1)	8 (1.25)	8.25 (1)	0.162
	Boys (single-sex)	54	8.29 (0.83)	8.25 (1)	8.5 (1)	8.25 (1)	
	Girls (single-sex)	43	7.92 (1.17)	8 (1)	8 (1)	7.75 (1)	

*Since the scores were non-normally distributed, the median has been reported as a central tendency measure and the IQR (in parentheses) as a measure of data dispersion. N, sample size; TSES, Teachers’ Sense of efficacy scale; IS, efficacy for instructional strategies; CM, efficacy for classroom management; SE, efficacy for student engagement.*

Furthermore, we found teachers of seventh grade had the lowest medians for the different self-efficacy factors. Nonetheless, after conducting the Kruskal-Wallis *H* tests, the only significant differences found among groups regarded SE scale: *H*(4) = 11.371, *p* = 0.023. Teachers in 7th grade displayed lower SE levels than teachers in 4th grade (*z* = −1.96, *p* = 0.025, *r* = 0.14), 6th grade (*z* = −1.93, *p* = 0.027, *r* = 0.14), and 8th grade (*z* = −2.36, *p* = 0.01, *r* = 0.17).

With respect to years of teaching experience, a positive correlation was found with teacher self-efficacy (*r* = 0.17, *p* = 0.02), IS (*r* = 0.15, *p* = 0.03), and CM (*r* = 0.16, *p* = 0.02), whereas SE subscale did not correlate with years of teaching. Table 5 shows the medians and ranks regarding five established groups (1–5 years, 6–10 years, 11–15 years, 16–20 years, and 21+ years; Dilekli and Tezci, 2020). After conducting the Kruskal-Wallis *H* tests, the only significant differences found among groups regarded CM scale: *H*(4) = 10.615, *p* = 0.031. Teachers with 16–20 years of teaching experience presented significantly higher levels of CM than teachers with 6–10 years of experience (*z* = −2.97, *p* = 0.015, *r* = 0.36).

Lastly, no significant differences were found regarding teacher self-efficacy based on employment at coeducational vs. single-sex schools. However, it is noteworthy that teachers employed at all-girls schools reported the lowest medians in all self-efficacy factors, whereas teachers at all-boys schools reported the highest values regarding self-efficacy (see Table 5).

## Discussion

This study aimed to examine the psychometric properties and obtain internal and external validity evidence supporting the use of the TSES in Spanish speakers while it is tested on a sample of Mexican teachers of private schools. Our results indicate that the Spanish TSES is a reliable instrument to measure and study teachers’ self-efficacy beliefs in the Mexican context. Internal consistencies of the scale and its subscales were good, ranging from 0.78 to 0.91, and were furthermore similar to those obtained in the original study: ranging from 0.81 to 0.90 (Tschannen-Moran and Woolfolk Hoy, 2001).

With reference to our first hypothesis, CFAs results indicate that the Spanish TSES has a three factor correlated structure, as originally proposed by Tschannen-Moran and Woolfolk Hoy (2001) for in-service teachers. As expected, the three factor correlated model showed better quality of fit than the one-factor model. However, RMSEA value (0.102) was higher than expected, and TLI value (0.886) was just below the critical threshold (0.08 and 0.9, respectively). Similar results have been reported by Klassen et al. (2009), who found RMSEA values were higher than expected for Cypriot (0.105) and Korean (0.134) teachers. Likewise, Valls et al. (2020) obtained a high RMSEA (0.097) and a low TLI (0.87) with Swiss teachers. According to Ninković and Knežević-Florić (2018): “the differences obtained by fit indices could be attributed to different response styles, specificities of the social and cultural context, and school conditions” (p. 84). Therefore, the RMSEA and TLI values indicated a certain discrepancy between the observed and expected values. It is worth noting that both indices are affected by sample size and may lead to false model rejections when the sample size is not adequate (Hu and Bentler, 1999). For this reason, it would be valuable that future studies conduct factor analyses of the TSES employing larger samples in order to assess the possible improvement of such goodness of fit indexes.

As to our second hypothesis regarding criterion validity evidence, the global construct of self-efficacy and its three factors showed positive and significant correlations with the JSC, as well as one of its subscales: job satisfaction with work environment. These results align with previous findings that suggest self-efficacy helps increase teacher job satisfaction in different educational contexts (Vieluf et al., 2013). In the present study, the classroom management factor displayed the highest correlations with work environment satisfaction, suggesting that when teachers perceived themselves as more capable of handling their class, they were more likely to feel satisfied with their job, and specifically, with their work environment. It is worth noting that the subscale of job satisfaction with profession did not correlate with teacher self-efficacy. These effects are manifested in a lower magnitude of the evidence of external validity between the TSES and job satisfaction in the sample of Mexican teachers. We must consider the negative asymmetric distribution of the scores of the job satisfaction with the profession subscale obtained in our sample, which in turn are manifested in low reliability. The latter increases the measurement error and is likely to attenuate the true correlations. Furthermore, although the TALIS 2018 report indicates a significant positive correlation between self-efficacy and job satisfaction, empirical evidence suggests that the final data corresponding to the job satisfaction survey was published with errors in the coding of its reversed items (Zakariya, 2020), which would call into question the objectivity of the published relations in the TALIS report, as well as it would require a new evaluation of its results. From a theoretical perspective, this may stem from the fact that satisfaction with the profession refers to more general aspects than capability beliefs, and rather seeks to determine whether or not a teacher would choose the teaching profession again if given the chance to go back in time and choose a career. Satisfaction with the work environment, however, focuses on a teacher’s contentment regarding their current employment at their given school.

Further analyses were performed to examine group differences proposed in hypothesis three regarding Mexican teachers’ self-efficacy. For this purpose, the literature typically uses parametric methods such as *t*-tests (for comparing two groups) and ANOVAs (for comparing more than two groups). However, after verifying that self-efficacy had a non-normal distribution, we used non-parametric tests that allowed a more valid data interpretation. Consequently, the Mann-Whitney *U* test and Kruskal-Wallis *H* test were used in this study. As some studies have previously found (Klassen and Chiu, 2010; Lumpe et al., 2012; Gulistan et al., 2017), male teachers appeared more confident in their capability to teach their students than female teachers. This difference is more clearly seen in the medians and ranks displayed by the teachers relative to the school model. In this way, as shown in Table 5, all-boys schools with only male teachers presented higher levels of self-efficacy in all factors, whereas all-girls schools with only female teachers obtained the lowest levels of self-efficacy in all factors. Meanwhile, coeducational schools presented intermediate values, with 82.7% of these teachers being female and 17.2% male.

Concerning self-efficacy and school grade, seventh-grade teachers appeared to have the lowest self-efficacy levels. However, the only significant difference found concerned the student engagement subscale, suggesting that seventh-grade teachers perceived themselves as less capable of engaging students in the learning process. Similarly, Backhoff Escudero and Pérez-Morán (2015) found in the Mexican TALIS report that secondary education teachers presented lower levels of self-efficacy regarding student engagement than primary teachers. On the international stage, Fives and Buehl (2009) noted that elementary teachers presented higher levels of efficacy for student engagement than teachers at secondary schools, suggesting a need for targeted professional development programs for secondary teachers regarding student engagement.

The present study showed a curvilinear relationship between self-efficacy and years of teaching experience, similar to the one found by Klassen and Chiu (2010). Teachers with 16–20 years of experience seem to have greater levels of self-efficacy than teachers with more or less years in the teaching profession. However, it is striking that teachers with 1–5 years in the teaching profession seem to have higher levels of self-efficacy than teachers with 6–10 and 11–15 years of teaching experience. These results led us to think that years of experience were not equally distributed in all school grades in this sample. To this respect, Table 6 illustrates how teaching experience is distributed across the different school grades (4th–8th grade). This table shows that the years of teaching experience in our sample were not evenly distributed regarding the school grade, which could be affecting the relationship between self-efficacy and teaching experience. I.e., most of the teachers with 1–5 years of experience are teaching in 4th grade, this may be the reason why beginner teachers in our sample appear to have higher levels of self-efficacy than middle experienced teachers. Future studies should include a more homogeneous distribution regarding teaching experience, so as to assess the effect of grade level taught on self-efficacy by controlling for teaching experience.

**TABLE 6:** Sample size and TSES scores regarding school grade and teaching experience.

**School grade**	**Teaching experience**					**N**	**TSES**
	**1–5 years**	**6–10 years**	**11–15 years**	**16–20 years**	**21 or more**		
4th	15	5	7	8	10	45	8.17 (0.91)
5th	2	8	8	5	16	39	8.17 (1)
6th	2	6	6	7	13	34	8.25 (0.75)
7th	6	12	8	3	5	34	7.92 (0.58)
8th	6	4	11	8	9	38	8.25 (1.08)
N	31	35	40	31	53	190	
TSES	8 (1)	7.83 (0.92)	7.88 (1.21)	8.33 (0.91)	8.25 (0.58)		

*Since the TSES scores were non-normally distributed, the median has been reported as a central tendency measure, and the IQR (in parentheses) as a measure of dispersion. N, sample size; TSES, Teachers’ Sense of Efficacy Scale.*

Lastly, it is worth nothing that the Mexican teachers who participated in this study showed relatively high levels of self-efficacy (as presented in Table 3). The means obtained in the original study ranged between 6.7 and 7.3 (Tschannen-Moran and Woolfolk Hoy, 2001) similar to those obtained in studies such as those of Ninković and Knežević-Florić (2018) and Valls et al. (2020); however, all means and medians from the present study were above 8 (on a scale rated from 1 to 9). These scores indicate that the majority of values obtained approach the upper limit of the scale used in its measurement, pointing to the possibility of a ceiling effect. A possible interpretation of this effect is that it could indicate a greater perception of self-efficacy beliefs in this sample of Mexican teachers in comparison with the rest of studies. Considering that the instrument has been tested in samples of different countries and that the response’ options cover the same range values as the original scale (e.g., from 1 = none to 9 = a great deal), we do not believe the validity of the score interpretability to be limited. Specifically, we believe that the ceiling effect could be due to response bias which could in turn be produced by social desirability or a distorted perception of the self-efficacy domain of the participants. A recent study has indicated inconsistencies between personal judgments and teacher performance in a small sample of 24 teachers from Monterrey (Cocca et al., 2018), suggesting a distorted perception of their in-class performance, which could drive to lower quality of the teaching-learning process. The ceiling effect could be of particular concern during the evaluation of self-efficacy in specific samples and in longitudinal studies, as it would decrease the likelihood that the instrument will accurately measure this particular domain (Roberts et al., 2001; Kuusinen, 2016). To better understand the ceiling effect we found on this sample, it might be useful to check the TSES scores in a larger sample which include public teachers.

Finally, the higher values obtained on this sample may be due to varying reasons: the use of a self-report questionnaire that reflects a social desirability bias (Organization for Economic Co-operation and Development [OECD], 2019a); the Likert scale, which ranges from 1 to 9 and may encourage overestimation (Valls et al., 2020), especially with Mexican teachers accustomed to scores ranging from 1 to 10; or the possible influence of cultural values like individualism and collectivism that have been detailed in previous studies (Vieluf et al., 2013; Fackler, 2018).

### Limitations and Future Directions

Although our results are encouraging, some limitations should be addressed. First, the size of the sample was relatively small, however the obtained results were in line with previous studies. Second, in order to compare self-efficacy beliefs within teachers from the same school but different educational levels, participating teachers were all from private schools; thus, the findings of this research should be considered in the context of these characteristics. Further research should be conducted to study the psychometric properties of the Spanish TSES while considering the diversity of contexts within Mexico. Specifically, it would be useful to understand the self-efficacy beliefs of teachers at public schools, which account for about 90% of enrollment in primary and secondary education in Mexico (Instituto Nacional para la Evaluación de la Educación [INEE], 2019).

The aim of this study was to validate evidence associated with the Spanish-TSES while maintaining the characteristics of the original validation sample (e.g., primary and secondary) as well as its internal structure and response options. Considering the differences in the short-TSES scores found between our sample and samples of other countries, the moderate fit of our factor model and the characteristics of the current sample (small sample of private school teachers), we recommend that future studies include larger and general (public and private) samples and test for alternative factor models, and contemplate different scale responses (e.g., a 5-point Likert scale), so that the evidence shown in the present study could be accepted or rejected.

Future studies in Mexico and Latin America would be of great help so as to better understand how teacher self-efficacy behaves in these countries. Perhaps including a social desirability questionnaire or adapting the responses to a Likert scale from 1 to 5, or 1 to 7, may be useful to obtain more accurate results. Longitudinal and qualitative studies may also help to delve into the different nuances of teacher self-efficacy and how these beliefs are developed throughout the teaching career in the Spanish-speaking context.

Finally, since the scale has been revised by Spanish and Mexican experts who ensured the neutrality of the language used, researchers are furthermore encouraged to use this version of the TSES in different Spanish-speaking countries. The consistent use of a reliable validated scale for measuring teachers’ self-efficacy would aid in promoting comparative studies on this topic. In this vein, further research should explore how cultural values in Spanish-speaking countries influence the ways in which teachers assess themselves as more or less capable of achieving proposed educational objectives.

## Data Availability Statement

The datasets presented in this article are not readily available because no consent was obtained from subjects to publicly share their pseudonymized data or to anonymize the data. Requests to access the datasets should be directed to FS-R (fsalas@alumni.unav.es).

## Ethics Statement

Ethical approval was obtained by the Research Ethics Committee of the University of Navarra (Project ID: 2020.042). Written informed consent for participation was not required for this study in accordance with the national legislation and the institutional requirements.

## Author Contributions

FS-R, SL, and MM designed the study. FS-R contributed to the data collection, data analysis, and writing. SL contributed to the conceptualization and supervision of the study. MM contributed to the data analysis. All authors revised the draft, made substantial contributions, and approved the final manuscript.

## Conflict of Interest

The authors declare that the research was conducted in the absence of any commercial or financial relationships that could be construed as a potential conflict of interest.

## Publisher’s Note

All claims expressed in this article are solely those of the authors and do not necessarily represent those of their affiliated organizations, or those of the publisher, the editors and the reviewers. Any product that may be evaluated in this article, or claim that may be made by its manufacturer, is not guaranteed or endorsed by the publisher.

## Abbreviations

TSES: Teachers’ Sense of Efficacy Scale
IS: instructional strategies
CM: classroom management
SE: student engagement
CFA: confirmatory factor analysis
JSC: job satisfaction composite scale
JSWE: job satisfaction with work environment
JSP: job satisfaction with profession.
