# Supplementary material for: Spanish Version of the Teachers’ Sense of Efficacy Scale: An Adaptation and Validation Study
Source: Front Psychol. 2021 Nov 11;12:714145. doi: 10.3389/fpsyg.2021.714145 (PMC8631807; doi:10.3389/fpsyg.2021.714145)
Supplement: Supplementary file 1 [file Data_Sheet_1.docx]

Supplementary Material

# Annex 1 – Spanish Version of the Teachers’ Sense of Efficacy Scale

**Escala de creencias de autoeficacia docente**

Instrucciones: Por favor, indica cuál es tu opinión acerca de cada pregunta. Para ello, escoge una respuesta de la siguiente escala, que va desde (1) “Nada/En absoluto” hasta (9) “Mucho/Muy bien”.

| 1 | 2 | 3 | 4 | 5 | 6 | 7 | 8 | 9 |
| --- | --- | --- | --- | --- | --- | --- | --- | --- |
| Nada /  En absoluto |  | Muy poco |  | Algo /  En alguna medida |  | Bastante |  | Mucho /  Muy bien |

Responde a cada pregunta teniendo en cuenta la capacidad, los recursos y las oportunidades que, desde tu posición laboral, tienes *actualmente* para llevar a cabo cada una de las siguientes cuestiones:

|  | Nada/En absoluto |  | Muy poco |  | Algo/En alguna medida |  | Bastante |  | Mucho/Muy bien |
| --- | --- | --- | --- | --- | --- | --- | --- | --- | --- |
| 1. ¿Cuánto puedes hacer para controlar el comportamiento disruptivo en el aula? | ① | ② | ③ | ④ | ⑤ | ⑥ | ⑦ | ⑧ | ⑨ |
| 1. ¿Cuánto puedes hacer para motivar a los alumnos que muestran un bajo interés en sus tareas escolares? | ① | ② | ③ | ④ | ⑤ | ⑥ | ⑦ | ⑧ | ⑨ |
| 1. ¿Cuánto puedes hacer para calmar a un alumno que se comporta de manera disruptiva o ruidosa? | ① | ② | ③ | ④ | ⑤ | ⑥ | ⑦ | ⑧ | ⑨ |
| 1. ¿Cuánto puedes hacer para ayudar a tus alumnos a valorar el aprendizaje? | ① | ② | ③ | ④ | ⑤ | ⑥ | ⑦ | ⑧ | ⑨ |
| 1. ¿En qué medida puedes formular buenas preguntas a tus alumnos? | ① | ② | ③ | ④ | ⑤ | ⑥ | ⑦ | ⑧ | ⑨ |
| 1. ¿Cuánto puedes hacer para que tus alumnos cumplan las normas en el aula? | ① | ② | ③ | ④ | ⑤ | ⑥ | ⑦ | ⑧ | ⑨ |
| 1. ¿Cuánto puedes hacer para que tus alumnos se crean capaces de realizar con éxito sus tareas escolares? | ① | ② | ③ | ④ | ⑤ | ⑥ | ⑦ | ⑧ | ⑨ |
| 1. ¿Hasta qué punto puedes establecer un sistema de gestión del aula con cada grupo de alumnos? | ① | ② | ③ | ④ | ⑤ | ⑥ | ⑦ | ⑧ | ⑨ |
| 1. ¿En qué medida puedes emplear estrategias de evaluación variadas? | ① | ② | ③ | ④ | ⑤ | ⑥ | ⑦ | ⑧ | ⑨ |
| 1. ¿En qué medida puedes proporcionar explicaciones o ejemplos alternativos cuando tus alumnos tienen dudas? | ① | ② | ③ | ④ | ⑤ | ⑥ | ⑦ | ⑧ | ⑨ |
| 1. ¿Cuánto puedes apoyar a las familias para que ayuden a sus hijos a ir bien en el colegio? | ① | ② | ③ | ④ | ⑤ | ⑥ | ⑦ | ⑧ | ⑨ |
| 1. ¿Hasta qué punto puedes llevar a la práctica estrategias docentes alternativas en el aula? | ① | ② | ③ | ④ | ⑤ | ⑥ | ⑦ | ⑧ | ⑨ |

# Annex 2 – Job Satisfaction Scale in Spanish

Nos gustaría saber cómo se siente en general con respecto a su trabajo. Indique su grado de acuerdo o desacuerdo con las siguientes afirmaciones.

| 1 | 2 | 3 | 4 |
| --- | --- | --- | --- |
| Totalmente en desacuerdo | En desacuerdo | De acuerdo | Totalmente de acuerdo |

| 1. Las ventajas de la profesión docente superan claramente las desventajas. | ① | ② | ③ | ④ |
| --- | --- | --- | --- | --- |
| 1. Si pudiera decidir de nuevo, volvería a elegir la profesión docente. | ① | ② | ③ | ④ |
| 1. Me gustaría cambiarme a otra escuela si eso fuera posible. | ① | ② | ③ | ④ |
| 1. Lamento haber decidido llegar a ser profesor/a. | ① | ② | ③ | ④ |
| 1. Disfruto trabajando en esta escuela. | ① | ② | ③ | ④ |
| 1. Me pregunto si habría sido mejor elegir otra profesión. | ① | ② | ③ | ④ |
| 1. Recomendaría esta escuela como un buen lugar para trabajar. | ① | ② | ③ | ④ |
| 1. En general, estoy satisfecho con mi trabajo. | ① | ② | ③ | ④ |
